# Quantitative comparison of the dynamic flow waveform changes in 12 ruptured and 29 unruptured ICA–ophthalmic artery aneurysms

**DOI:** 10.1007/s00234-012-1108-7

**Published:** 2013-02-27

**Authors:** Aichi Chien, James Sayre, Fernando Viñuela

**Affiliations:** 1 Division of Interventional Neuroradiology, David Geffen School of Medicine at UCLA, 10833 LeConte Avenue, Box 951721, Los Angeles, CA 90095 USA; 2 Department of Biostatistics, School of Public Health, University of California, Los Angeles, CA 90095 USA

**Keywords:** Aneurysm, Subarachnoid hemorrhage, Hemodynamics, Flow waveform

## Abstract

**Introduction:**

Studies have reported a correlation between blood flow dynamics in the cardiac cycle and vascular diseases, but research to analyze the dynamic changes of flow in cerebral aneurysms is limited. This quantitative study investigates the temporal changes in flow during a cardiac cycle (flow waveform) in different regions of aneurysms and their association with aneurysm rupture.

**Methods:**

Twelve ruptured and 29 unruptured aneurysms from the internal carotid artery–ophthalmic artery segment were studied. Patient-specific aneurysm data were implemented to simulate blood flow. The temporal flow changes at different regions of the aneurysm were recorded to compare the flow waveforms.

**Results:**

In more than 60 % of the cases, peak flow in the aneurysm sac occurred after peak flow in the artery. Flow rate varied among cases and no correlation with rupture, aneurysm flow rate, and aneurysm size was found. Higher pulsatility within aneurysm sacs was found when comparing with the parent artery (*P* < 0.001). Pulsatility was high throughout ruptured aneurysms, but increased from neck to dome in unruptured ones (*P* = 0.021). Significant changes between inflow and outflow flow profile were found in unruptured aneurysms (*P* = 0.023), but not in ruptured aneurysms.

**Conclusion:**

Quantitative analysis which considers temporal blood flow changes appears to provide additional information which is not apparent from aneurysmal flow at a single time point (i.e., peak of systole). By considering the flow waveform throughout the cardiac cycle, statistically significant differences were found between ruptured and unruptured cases — for flow profile, pulsatility and timing of peak flow.

## Introduction

Dynamic blood flow changes in the artery are known to be associated with vascular disease [1, 2]. Blood flow waveform changes in large arteries have been reported in patients with impaired vessel wall function. Studies have found that in large vessels the flow waveform is greatly influenced by the vessel compliance and by the reflected wave caused by the geometric resistance of the lumen shape [3–5]. However, existing research about the characteristics of flow waveform in smaller vessels, such as in patients with cerebral vascular diseases, is limited.

Measurements of cerebral blood flow waveforms in patients are challenging due to the small vessel dimensions and procedure risks [6]. Recently, researchers applied a magnetic resonance imaging-derived method and revealed the waveform characteristics of cerebral vessels in healthy subjects. Studies found that the flow waveform varies within normal branches of the cerebral vasculature, waveform changes in cerebral vessels are likely related to the vascular shape, and they may have protective effects for cerebral vasculature [7, 8].

A cerebral aneurysm is a localized, bulging vessel malformation which can lead to hemorrhagic stroke when it ruptures [9]. The mechanisms of aneurysm rupture are currently unknown and have been found to relate to aneurysmal blood flow [10–12]. Recently, researchers found that in the presence of an aneurysm, the blood flow waveform changes in both the aneurysm and artery [13]. However, there is a lack of studies comparing the flow waveforms in clinical ruptured and unruptured aneurysms.

This research aims to investigate the dynamics of flow in ruptured and unruptured cerebral aneurysms. As previous research has characterized flow changes in different segments of the cerebral artery and different aneurysm locations [7, 8, 14], to minimize waveform changes due to location, we analyzed a group of aneurysms specifically located at the segment of internal carotid artery connecting with the ophthalmic artery. We quantitatively compared the temporal and spatial changes of flow waveform in different regions of the aneurysm and sought to find the characteristics of flow waveform in ruptured and unruptured groups.

## Material and methods

### Brain aneurysm cases

This study was approved by Institutional Review Board. Patients with brain aneurysms at the internal carotid artery–ophthalmic artery segment were selected consecutively from the aneurysm database which recorded brain aneurysm cases treated from January 2004 to March 2011 in the Division of Interventional Neuroradiology at the UCLA Medical Center.

A total of 41 aneurysms (12 ruptured and 29 unruptured) from 40 female and one male patient were included in this study. The aneurysm average size (largest diameter of aneurysm sac) was 9.5 mm (11.8and 8.5 mm for ruptured and unruptured cases, respectively). The average aneurysm sac to neck ratio was 2.1 (2.6 and 2.0 for ruptured and unruptured cases, respectively). Table 1 summarizes the detail of the cases. Clinical cerebral angiograms acquired before aneurysm embolization treatment were collected for the aneurysmal flow analysis. Aneurysms were imaged using 3D rotational angiography (3DRA, Philip Medical System, Best, the Netherlands) and 3D reconstruction of aneurysm geometry was performed using an Integris workstation (Philips Medical System) [15].

**Table 1:** Summary of aneurysm cases

Characteristics	Ruptured aneurysm	Unruptured aneurysm	Summary
Number of aneurysms	12	29	41
Patient age (years)			
Mean	58	56	57
Range	25–77	17–75	17–77
Patient gender (no. of patients)			
Female	12	28	40
Male	0	1	1
Size of aneurysm (no. of aneurysms)			
<7 mm	4	10	14
7–12 mm	2	16	18
>12 mm	6	3	9
Largest diameter of aneurysm sac (mm)			
Mean	11.8	8.5	9.5
Range	3.7–23.5	3.8–19.5	3.7–23.5
Size of Aneurysm neck (mm)			
Mean	4.5	4.4	4.4
Range	2.7–7.0	2.5–10.0	2.5–10.0
Aneurysm sac/neck ratio			
Mean	2.6	2.0	2.1
Range	1.1–4.9	1.3–3.8	1.1–4.9

### Computation of the aneurysm flow waveform

Previously developed, patient-specific computational flow analysis was applied to study the aneurysmal blood flow [10, 16]. Since the simulation inflow conditions may influence the aneurysmal flow, all of the patient-specific aneurysm models were reconstructed beginning with the ICA cavernous region to ensure the inflow condition is applied the same way for all the aneurysms and that the inflow boundary is distant from the aneurysm area [13]. Blood flow was modeled as an incompressible Newtonian fluid using the unsteady 3D Navier–Stokes equations. The pulsatile flow profile was applied by implementing the ICA flow profile acquired from a healthy subject with phase-contrast MR imaging (Signa 1.5-T scanner; GE Healthcare, Waukesha, WI). The flow profiles were prescribed using the Womersley solution for fully developed pulsatile flow in a rigid straight tube and scaled according to the cross-sectional area of the inflow vessels for each model. For the outflow condition, traction-free boundary conditions with the same pressure level were applied. Rigid and no-slip boundary conditions were assumed at the vessel walls. The blood attenuation was set as 1.0 g/cm^3^ and the viscosity was 0.04 poise. Numerical solutions of the Navier–Stokes equations were obtained using a fully implicit finite-element formulation.

**Fig. 1:**
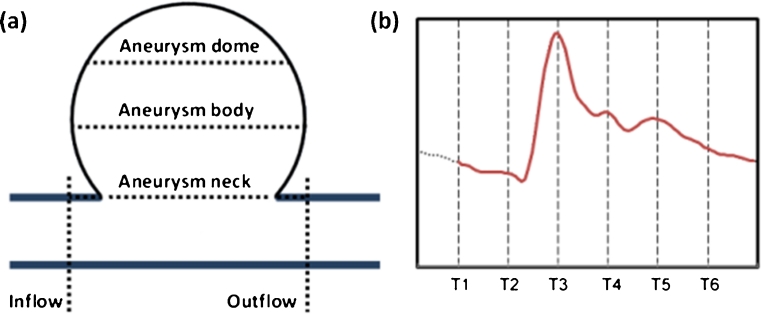
Left is the schematic representation of aneurysm regions which were specified to compare the flow changes. For each aneurysm region, the flow data was collected at six different time points of a cardiac cycle (*right*) for the flow waveform analysis

To ensure the numerical solution was stabilized, a total of two cardiac cycles were computed by using 100 steps per cycle. The flow results from the second cardiac cycle were used to analyze the dynamic changes of aneurysm flow. For each aneurysm, flow data was collected from five regions of the aneurysm at six different time points (T1–T6) which evenly divided the cardiac cycle into six intervals, with T3 at the timing of peak systole. Specifically, we recorded the volumetric flow rate at locations of aneurysmal inflow, aneurysmal outflow, aneurysm neck, aneurysm body and aneurysm dome following the same procedure as the previously reported quantitative analysis technique — evenly dividing the aneurysm sac into three segments to collect flow data (neck, body and dome) [14]. Schematic representation of the data collection at regions of an aneurysm and time points in the cardiac cycle are shown in Fig. 1. We compared the flow waveform from aneurysm regions by analyzing the flow profile in a cardiac cycle (FP), the timing of peak flow (TPF), and the flow pulsatility index (PI). PI was obtained using Eq. 1.

1 $$ \mathrm{PI}\frac{{\mathrm{miximun}\;\mathrm{flow}\;\mathrm{rate}\;\text{-}\;\mathrm{minimum}\;\mathrm{flow}\;\mathrm{rate}}}{{\mathrm{mean}\;\mathrm{flow}\;\mathrm{rate}}} $$

### Statistical analysis

The multivariate Hotelling’s T^2^ test was used to compare the FP, TPF, and PI of the blood flow waveform between ruptured and unruptured cases. Individual paired comparisons were performed using paired *t*-tests. Fisher’s exact test was used to assess proportion shifts between ruptured and unruptured cases. Level of significance was set at 5 %. Stata 11 and IBM SPSS 20 statistical software were used to perform the statistical analysis.

## Results

**Fig. 2:**
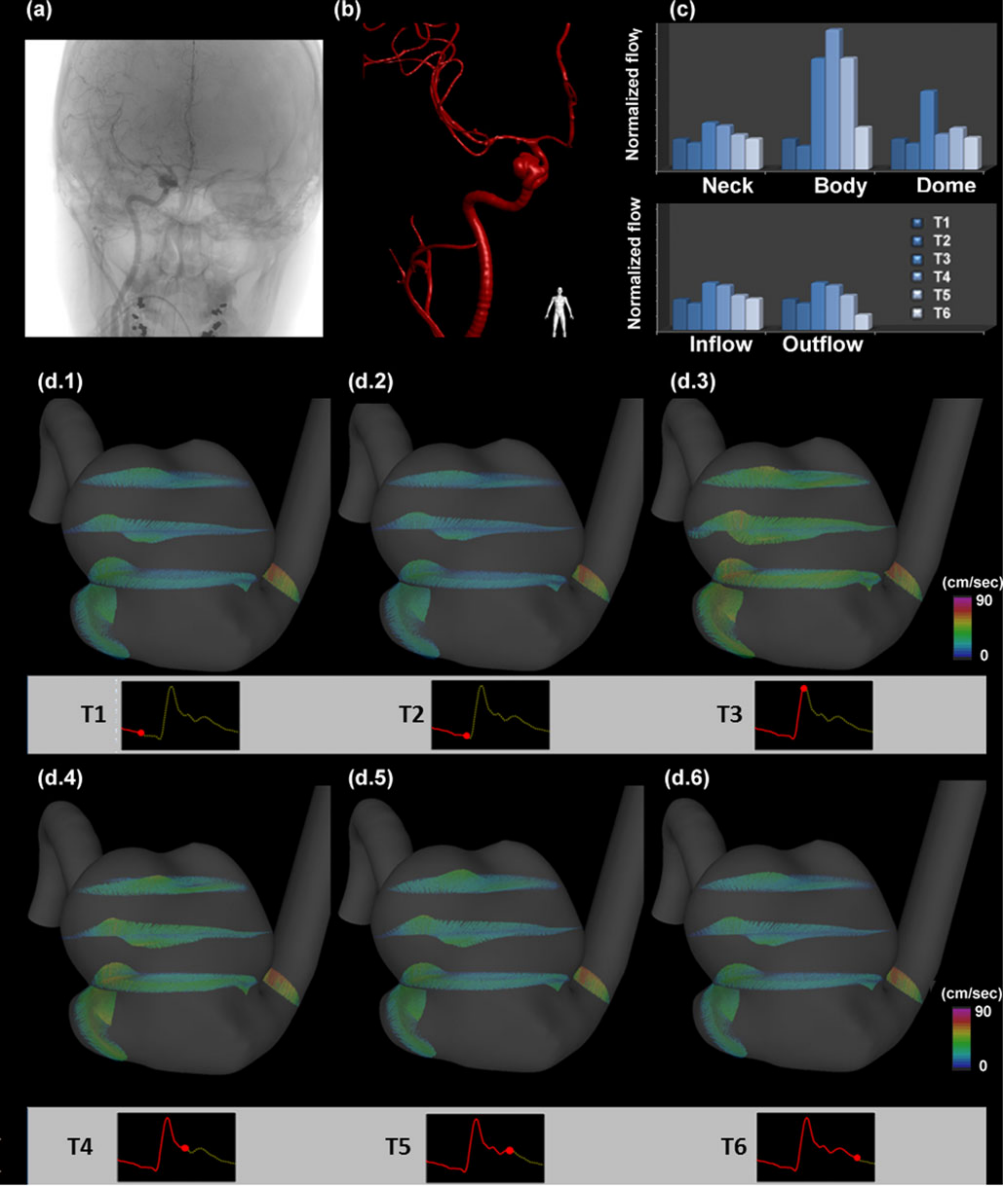
Flow changes in time at different regions of an aneurysm. **a** Original clinical angiogram to image the aneurysm. **b** 3D reconstruction of the blood vessels and the aneurysm. **c** Flow changes at different regions of the aneurysm (normalized flow using T1 flow rate as baseline) (**d.1**–**d.6**) the flow vector plots at regions of the aneurysm at corresponding times

Representative flow changes in an aneurysm at different time points are shown in Fig. 2. The waveform changes within the aneurysm sac can be observed in Fig. 2c. In the aneurysm body and dome regions, the flow changes from T1 to T6 do not follow the same profile as in the inflow, outflow and neck regions of the aneurysm. Also, the TPF in the aneurysm body is delayed from time T3 to T4. Figure 2d presents the flow vector field and illustrates changes of flow in aneurysm regions.

**Fig. 3:**
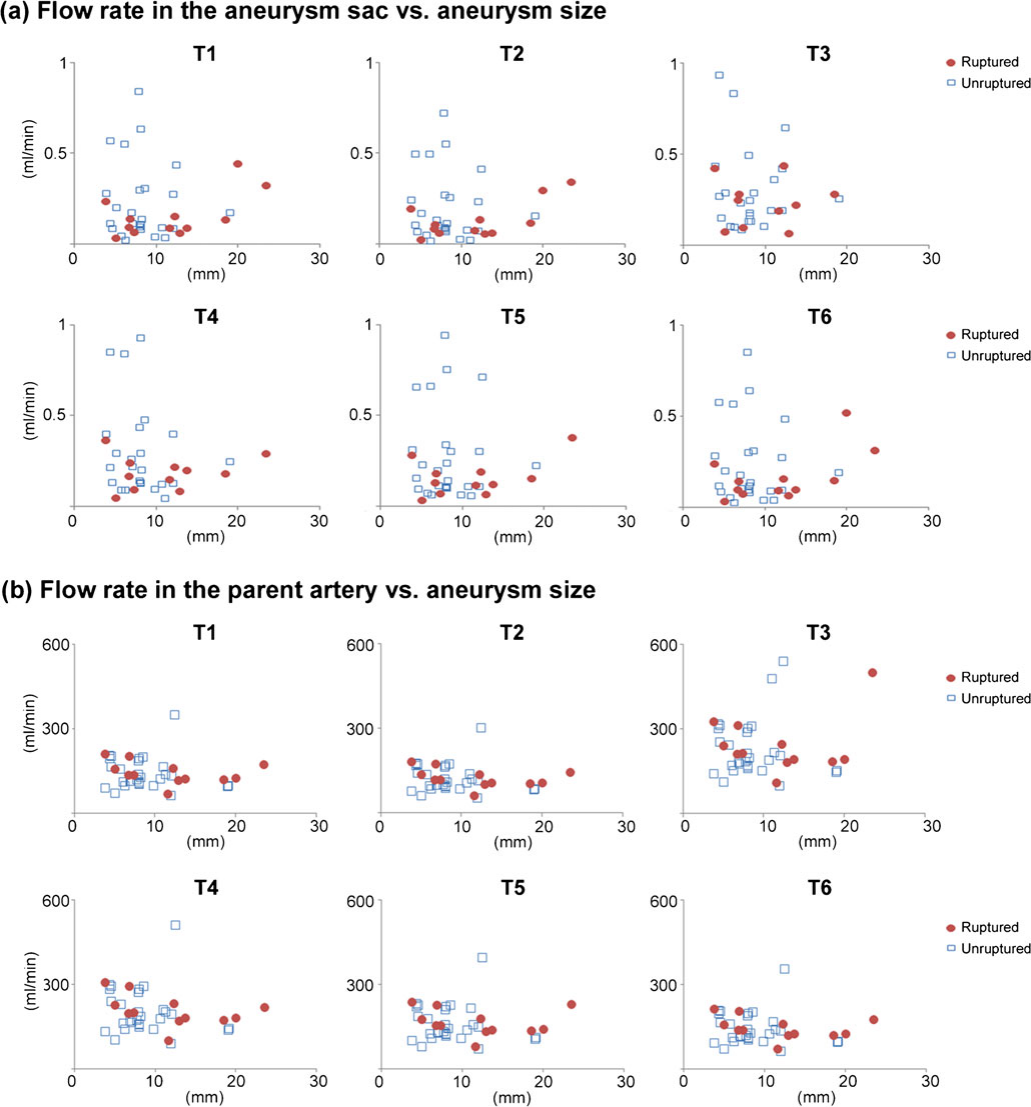
The flow rate in the aneurysm sacs and parent artery at different time points for 12 ruptured and 29 unruptured aneurysms. **a** Flow rate in the aneurysm sacs and **b** flow rate in the parent artery do not correlate with aneurysm size in all the time points of cardiac cycle

**Fig. 4:**
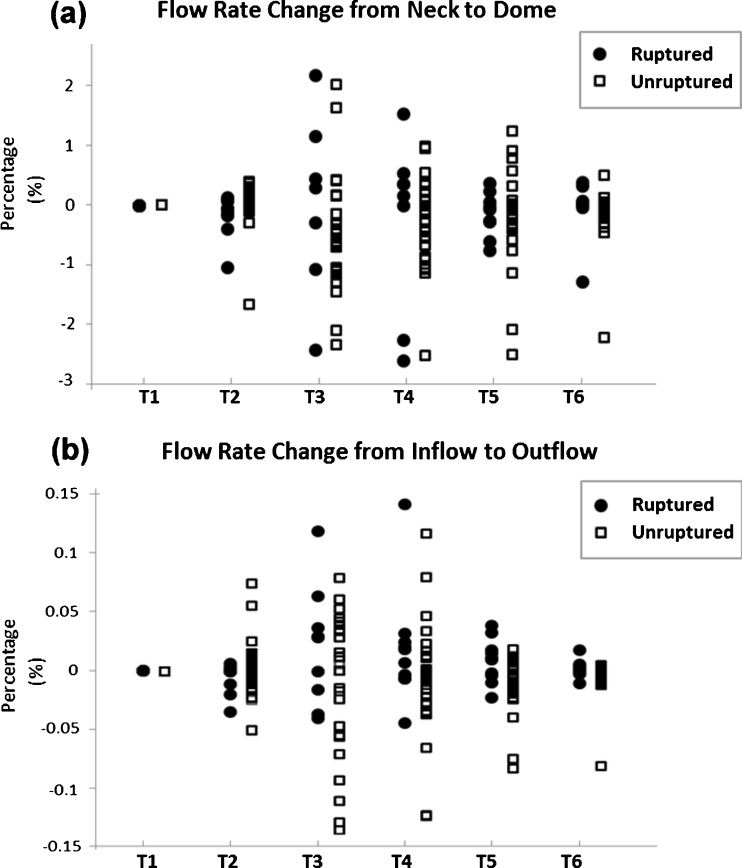
Flow rate change over a cardiac cycle in ruptured (*circles*) and unruptured (*rectangles*) aneurysms with respect to the flow rate at T1. Positive sign shows flow increases and negative sign shows flow decreases. **a** The flow from the neck to dome shows ruptured and unruptured aneurysms had the same trend of flow changes. **b** The flow from inflow to outflow shows ruptured aneurysms tended to have increased outflow; however, unruptured aneurysms tended to have decreased outflow

The average flow rate in the aneurysm sac and parent artery at different time points is reported in Fig. 3. Aneurysm flow rate is not correlated with aneurysm size at all the time points of the cardiac cycle. No difference was found between ruptured and unruptured groups when comparing the flow rate at individual time points. We further compared the flow rate changes within the aneurysm at each time point (Fig. 4). We found flow rate changes from the neck to the dome did not differ between ruptured and unruptured cases; however, when comparing the difference between inflow and outflow, we found that ruptured aneurysms tended to have increased outflow over the cardiac cycle.

We also observed that 25 of 41 cases had the TPF within the aneurysms desynchronized with the TPF in the inflow and outflow regions, as shown in Fig. 2c. While the TPF were all at T3 for inflow and outflow regions in all aneurysm cases, 25 aneurysms had TPF delayed to T4 in at least one of the neck, body and dome regions — 22 cases are unruptured aneurysms and three cases are ruptured aneurysms. TPF within the aneurysm sacs were significantly different between ruptured and unruptured cases (*P* = 0.004).

We also compared FP over the cardiac cycle and found significant FP change from the aneurysm neck to the aneurysm dome in all cases (*P* = 0.005). The change of FP within the aneurysm was not statistically different between ruptured and unruptured groups. Significant changes between the inflow and outflow FP were found in unruptured cases (*P* = 0.023). This trend of changes in inflow and outflow FP was not found in ruptured aneurysms (*P* = 0.277).

**Fig. 5:**
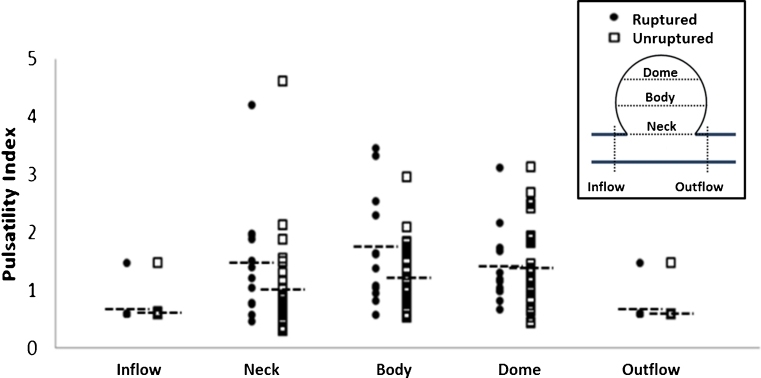
Pulsatility index in different regions of ruptured (*circles*) and unruptured (*rectangles*) aneurysms. For both ruptured and unruptured aneurysms, PI is higher in the aneurysm neck, body and dome comparing with inflow and outflow regions. However, ruptured cases had overall higher PI than unruptured cases

Study of the flow pulsatility showed that it increased in the aneurysm sac compared to the vessel (at the inflow and outflow regions) in both ruptured and unruptured aneurysms (*P* < 0.001) (Fig. 5). High PI was found within the entire ruptured aneurysm sacs; however, the PI gradually increased from the aneurysm neck to dome in unruptured aneurysm sacs (*P* = 0.021). No statistical difference was found between PI at inflow and PI at outflow (*P* = 0.483 in ruptured cases; *P* = 0.269 in unruptured cases).

## Discussion

Studies have reported that the blood flow waveform changes in large vessels and the waveform can be useful information to study vascular disease. Recently, researchers found that the flow waveform also changes in cerebral arteries among healthy individuals and suggested that the waveform changes in the cerebral vascular tree are related to vessel shape [7, 8]. Cerebral aneurysms which present localized changes in vessel shape are known to influence the blood flow at the site of malformation [17, 18]. However, a study investigating the dynamics of flow waveform changes between ruptured and unruptured aneurysms has not been reported.

It was previously reported that unstable flow can occur within an aneurysm after the peak of systolic flow [19]. In this study, we also found that some aneurysms (especially unruptured aneurysms) have peak flow (TPF) in later cardiac phases. These delayed TPF may result from the unstable circulating flow within an aneurysm. Therefore, our findings suggest that instead of only analyzing the flow at the timing of peak systole, studies may need to incorporate more time points for the comparison of aneurysm flow properties (including the flow characteristics and wall shear stress) to accurately identify and analyze the influence of peak flow to aneurysm rupture [10, 12, 16].

In the analysis of flow profile, we found that the inflow and outflow FP are different between ruptured and unruptured aneurysm cases. As reported by Baek et al. [13], the presence of aneurysms can cause pulsatile blood flow oscillations in the aneurysm and nearby artery. The different inflow and outflow FP relationship in ruptured and unruptured aneurysms suggests that the mechanism of flow oscillation may not be the same in all aneurysms. Further research is needed to study the relationship of flow wave changes with aneurysm rupture.

In both ruptured and unruptured cases, we found that PI was significantly higher within aneurysm sacs. The same observation that PI was higher in the aneurysms than in the artery was reported by Benndorf et al. in the clinical cases that they studied using Doppler guidewire to measure flow in aneurysms. We also found that the high PI was distributed throughout the ruptured aneurysm sacs, while it gradually increased from the neck to the dome in unruptured aneurysm sacs. As in the normal carotid tree the pulsatility decreases along the artery [7, 8], the sudden increase of high PI within ruptured aneurysm sacs suggests that the pulsatility of flow may have important influence in the mechanism of aneurysm rupture.

Although significant differences were found between inflow and outflow FP, we did not find differences in the PI at the inflow and outflow regions. This might be because the PI was calculated based on normalizing the difference of maximum and minimum flow rate with the mean flow rate (Eq. 1), and the flow differences between inflow and outflow may not occur at the maximum or minimum flow rate.

As in previous international reports on unruptured aneurysms, the majority of the cases in this study were unruptured aneurysms and the patients were predominantly female [20]. Although a comparable number of ruptured and unruptured aneurysms is essential to analyze flow differences between two groups, one of the major limitations in hemodynamic research is the rarity of data for ruptured aneurysm cases. To the best of our knowledge, the present research is the first hemodynamic study which analyzes the flow difference between ruptured and unruptured aneurysms with a comparable number of cases from a single aneurysm location [12].

### Limitations

Because ruptured and unruptured aneurysms may have different wall elasticity, due to factors such as wall spasm and surrounding hematoma, and the patient-specific aneurysm wall properties for individual patients are not available, in the current study, rigid wall properties were assumed for the hemodynamic simulation. As previous studies have shown that incorporating the aneurysm wall properties in the hemodynamic simulation changes the aneurysmal TPF to a later cardiac phase, the current finding additionally suggests that morphology may also cause a shift of TPF in the aneurysm [21].

Because this was a retrospective study, and obtaining individual patient flow profile was not part of common treatment protocol, the current flow simulation utilized the flow profile obtained from a healthy subject’s flow curve. However, in the future, incorporating patient-specific flow profiles obtained from phase-contrast magnetic resonance angiography into the simulation will be valuable to improve the patient-specific simulation methodology [22]. Future development of flow measurement technology to obtain flow data directly will be helpful to further improve the spatial and temporal accuracy of the flow simulation and allow detailed comparison of the flow waveform changes in aneurysms [18].

To systematically compare the aneurysm flow in a variety of aneurysm morphologies, we implemented a data collection method which divided the aneurysm sac into three segments— neck, body and dome. Although this quantitative hemodynamic analysis approach allows consistent comparison across various shapes and locations of aneurysms and has been verified and compared with other techniques, because some aneurysm necks are difficult to define with a plane, the data collection at the aneurysm neck region is still not ideal [14]. Future development of methods to study aneurysm flow at the aneurysm neck region may further improve the quantitative analysis.

## Conclusion

Quantitative analysis of temporal blood flow changes appears to provide additional information which is not apparent from aneurysmal flow at a single point in time. Analyzing a comparable number of similarly sized, ruptured and unruptured aneurysms from the ICA, we found that the flow rate at individual time points did not correlate with aneurysm size or rupture. However, blood flow waveform characteristics were found to differ between ruptured and unruptured aneurysms. Significant differences between ruptured and unruptured cases were found for flow profile, pulsatility, and timing of the peak flow, which were hidden when the comparison was performed based on an individual time point.

## Abbreviations

ICA: Internal carotid artery
FP: Flow profile
TPF: Timing of peak flow
PI: Pulsatility index
